# Vertical dynamic visual acuity is significantly lower than horizontal dynamic visual acuity

**DOI:** 10.1038/s41598-023-48292-1

**Published:** 2023-11-28

**Authors:** Aoi Tachihara, Zu Soh, Tomohiko Mizuguchi, Akihiko Kandori, Seiji Hama, Toshio Tsuji

**Affiliations:** 1 Electrical, Systems, and Control Engineering Program, Graduate School of Advanced Science and Engineering, Hiroshima University, Higashi-Hiroshima, Hiroshima 739-8527 Japan; 2 New Business Producing Division, Maxell Ltd., Yokohama, 240-0005 Japan; 3 Center for Exploratory Research, Research and Development Group, Hitachi. Ltd., Tokyo, 185-8601 Japan; 4 Department of Rehabilitation, Hibino Hospital, Hiroshima, 731-3164 Japan; 5 Department of Neurosurgery, Center for Brain, Mind and KANSEI Sciences Research, Hiroshima University, Hiroshima, 734-8553 Japan

**Keywords:** Health care, Health occupations, Biomedical engineering

## Abstract

Dynamic visual acuity (DVA) is crucial for the perception of moving objects. While traditional DVA assessment tools predominantly focus on horizontal movements, the evaluation of vertical DVA remains unstandardized. Consequently, the disparities between vertical and horizontal DVAs are yet to be thoroughly investigated. Therefore, we designed a system capable of conducting multidirectional DVA tests and eye movement measurements. During the experiments, the participants identified the gap direction of the Landolt-C ring moving either horizontally or vertically. The speed of movement decelerated from its maximum as a high-speed infrared camera captured the pupil movements of the left eye at 500 fps. We conducted tests on 15 healthy university students (aged $22.7\pm 1.2$ years) and measured vertical and horizontal DVAs five times each. DVA was deduced from the Landolt-C ring speed with accurate gap direction responses, and eye movement was assessed based on the total gaze movement distance. The results revealed superior DVA and eye movement in the horizontal direction compared with the vertical direction ($p < 0.001$). This highlights the anisotropic characteristics of DVA and eye movement. The proposed system has the potential for multidirectional dynamic vision evaluation and training in clinical scenarios.

## Introduction

Dynamic vision has been actively studied in sports science^1–3^ because most sports require the eyes to track fast-moving objects^4^. In general, athletes have better dynamic vision than non-athletes^5–8^. Dynamic visual acuity (DVA) is defined as the ability to discriminate details of an object during relative movement between the object and the observer^9^, occurring at the equidistance between the object and the eyes^7,10^. According to the sports vision pyramid^11^, DVA is the most fundamental dynamic vision ability^12^. Specifically, it is a monocular sensory process that serves as the basis for the subsequent process levels, such as binocular sensory perception and visual integration. This contribution highlights the importance of evaluating DVA.

Most DVA experiments tested the ability to identify an optotype moving horizontally^13–16^. Several studies have evaluated eye movements during DVA tests, revealing the importance of the ability of the eyes to track an optotype^17,18^. Compared with the horizontal DVA, the vertical DVA has not been adequately studied. A previous vertical DVA test presented a static visual target on a computer screen while moving the participant’s head vertically^19–26^. Employing a protocol that generates relative movement between the optotype and observer is indeed a valid method for measuring DVA; however, this type of method includes effects of the vestibulo-ocular reflex, and is incomparable to horizontal DVA measured by moving the optotype^27^. The horizontal DVA test uses a circular screen to project optotypes, whereas the vertical DVA test uses a flat computer screen. This difference in screen shape renders the comparison of vertical and horizontal DVA difficult. Therefore, it was considered more appropriate to present an optotype on a circular screen to test both vertical and horizontal DVA. Among the commercially available DVA test devices^7,28,29^, the Ishigaki-type DVA test device is the most frequently used^30^. This device presents a Landolt-C ring moving in the horizontal direction on a circular screen placed in front of the participant and evaluates DVA by the movement speed at which the participant could recognize the gap direction. However, the Ishigakitype device cannot present a vertically moving optotype, and there is no standard device for performing both horizontal and vertical DVA tests; hence, the directional characteristics of DVA have not been fully studied.

Therefore, this study aimed to investigate the differences in vertical and horizontal DVAs and related eye movements. To this end, we developed a measurement system that combines a multidirectional DVA device using a circular screen, similar to the Ishigaki-type device, with a high-speed infrared camera that records the eye and tracks the pupil movements. The developed device presents a moving optotype using a rotating mirror, reflecting a Landolt-C ring image generated by a projector and projecting the image onto a screen. The projector, mirror, and screen can be rotated as a single unit to represent the optotype moving horizontally and vertically. Using this system, we performed vertical and horizontal DVA tests on 15 participants, compared their DVA performances, and analyzed their eye movements.

**Figure 1:**
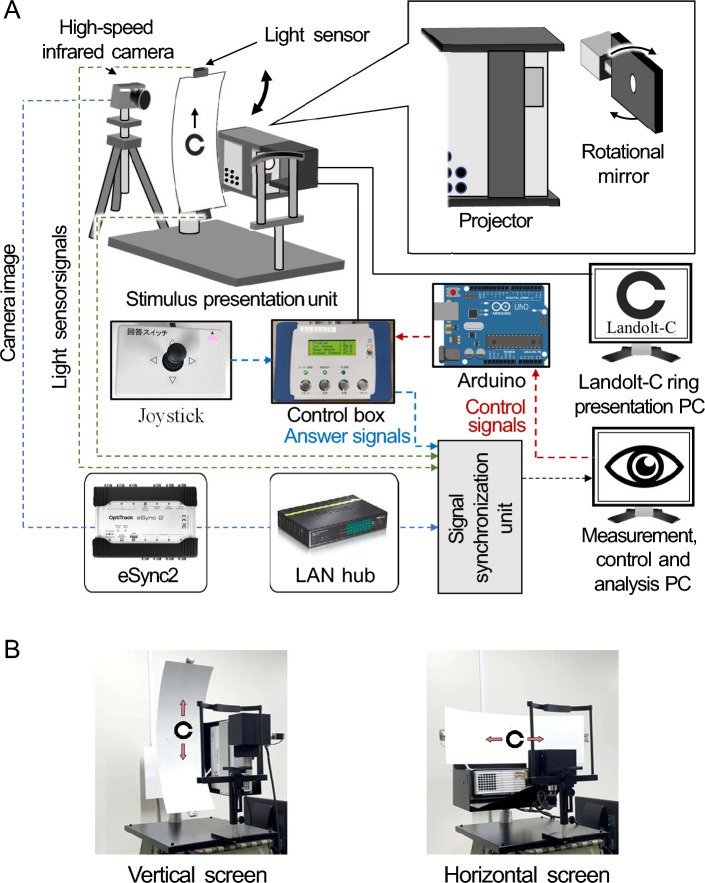
Multidirectional DVA test and eye movement measurement system. (**A**) Illustrates an overview of the system configuration. The solid and dashed lines represent control and measured signals, respectively. The circular screen, projector, and rotational mirror can be rotated together, enabling both vertical and horizontal DVAs tests. (**B**) Displays images of the developed system with the circular screen fixed in both vertical and horizontal orientations.

## Materials and methods

### Participants

The participants were 15 healthy university students ($22.7\pm 1.2$ years). Their mean static visual acuity, measured in decimal notation, was 0.67 (among all participants, seven participants had values between 0.1–0.5, and eight participants had values between 0.6–1.5). The participants were not allowed to wear glasses to help accurately capture pupil movements using a camera, which resulted in reduced visual acuity for some of them. Participants with low static visual acuity were included to validate the adaptability of the proposed system to a broad range of patients anticipated in the clinical practice. The Ethical Review Committee approved all research experiments at Hiroshima University (Permission No. E-725-5, E2018-1554-02), and informed consent was obtained from all participants. The study was conducted in accordance with the principles of the Declaration of Helsinki.

### Proposed system

Figure 1 illustrates the proposed multidirectional DVA test system. The system comprises a stimulus presentation unit, devices that measure DVA and eye movements, and a signal synchronization unit. In this section, we describe the components of the proposed system.

The stimulus presentation unit (upper left side of Fig. 1) consisted of a chin rest for fixing the participant’s head, a circular screen (viewing angle: $90.2 \times 28.6$ degrees) set 40 cm in front of the chin rest, a projector connected to a PC for presenting a Landolt-C ring, and a motor-driven rotational mirror controlled by a microcomputer. The height of the chin rest was adjusted according to each participant. The projector refresh rate was set to 120 Hz. The control algorithm of the digital micromirror devices installed in the projector was modified to present a flickerless image, wherein the white pixels of the Landolt-C ring were fixed to ON and the black pixels were fixed to OFF. With this configuration, we can reproduce the same conditions for Landolt-C ring presentation as in the Ishigaki-type DVA test device.

The Landolt-C ring image from the projector is reflected by a mirror (upper right of Fig. 1), and the motor rotates the mirror so that the Landolt-C ring moves around the rotational axis of the motor. When the reflection direction of the mirror meets the screen, the Landolt-C ring moves along the long axis. The circular screen, projector, and rotational mirror can be rotated together, allowing the display of a vertically or horizontally moving Landolt-C ring.

The measurement unit consisted of a response device, two light sensors, and a high-speed infrared camera. The participants used the response device to indicate the gap direction of the displayed Landolt-C ring. Light sensors were installed at both center ends of the circular screen and used to detect when the Landolt-C ring entered and exited the screen. A high-speed infrared camera (Prime Color FS, NaturalPoint, Oregon, USA), installed behind the circular screen, captured images of the participant’s left eyeball at a frame rate of 500 fps and a resolution of $960 \times 540$ pixels to track pupil movement.

**Figure 2:**
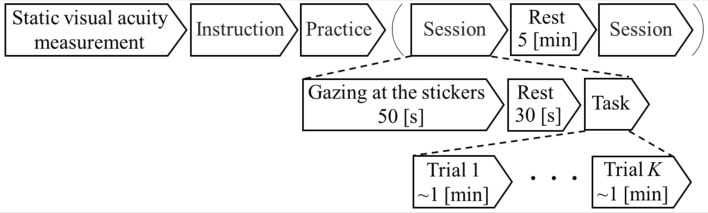
DVA test protocol. The procedures in the parentheses are repeated twice for different DVA test directions (vertical or horizontal) with a 5-min break in between.

**Figure 3:**
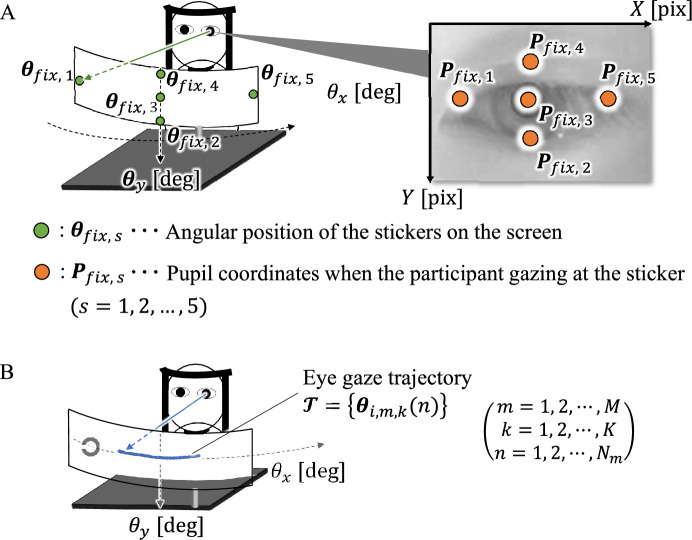
Calculation of the eye gaze position. (**A**) Illustrates the participant gazing at the position of stickers attached on the screen and the participant’s eye captured by the high-speed infrared camera. The orange circles indicate the pupil position when the participant’s gazing at the corresponding sticker (green circle) on the screen. (**B**) Illustrates an example of projecting a pupil trajectory to angular positions on the screen.

### Experimental protocol

Figure 2 explains the experimental protocol. Static visual acuity was measured using a Landolt-C ring chart. The room lighting and projector brightness were adjusted beforehand, so that the optotype was clearly visible. The participants were instructed to stand upright, facing the screen, with their chins secured to a chin rest that was adjusted to their height. The participants were advised to keep their heads stationary throughout the test. During the DVA test, the participants were directed to identify the gap direction of the Landolt-C ring quickly and accurately and to respond using the response device.

Before beginning the DVA test session, the average pupil positions were measured while gazing at the five stickers, as shown in Fig. 3A. The measured pupil positions were used to calibrate the gazing trajectories in the subsequent analysis phase. After resting for 30 s, two measurement sessions were performed, with either a vertical or horizontal DVA task assigned. A 5-min break was taken between measurement sessions. Each measurement session consisted of multiple trials, with one trial encompassing the presentation of the Landolt-C ring to the participant’s response. An adaptation of the psychophysical method of limits was employed in the experimental procedure. During the trial, the Landolt-C ring underwent a controlled decrease in rotational speed, slowing down from a maximum of 600 $^\circ$/s to a minimum of 120 $^\circ$/s, and decelerating incrementally by approximately 12 $^\circ$/s per rotation. The participants were asked to perform a four alternative forced choice procedure, specifically choosing the gap orientation (up, down, left, or right), using the response device. The mirror’s rotation was halted upon completing the rotation during which the participant responded. The rotational speed at which the participant answered correctly was registered as an index for DVA evaluation. This process was repeated in successive trials until five correct answers were obtained, ensuring the consistency and reliability of the measurement. To maintain randomness and reduce potential bias during the test, the traveling direction of the Landolt-C ring was alternated. In the vertical DVA test, the direction was changed from top to bottom or vice versa, whereas in the horizontal DVA test, it was altered from left to right or vice versa. This directional change was randomized for each trial and the participants were not notified in advance. By employing a systematic reduction in rotational speed and recording the corresponding correct responses, this method sought to ascertain the maximum speed at which participants could discern the gap orientation.

### Analysis

We used the markerless tracking software DeepLabCut^31^ to track the eye-gaze trajectories. The measured pupil coordinates were projected onto the angular coordinates of the screen using an affine transformation^32^. Using the pupil positions gazing at stickers, the parameters of the affine transformation were estimated by mapping the mean pupil positions $\varvec{P}_{fix, s}(s = 1, 2, \ldots , 5)$ onto the corresponding sticker positions $\varvec{\theta }_{fix, s}(s = 1, 2,\ldots , 5)$, as shown in Fig. 3A. Consider an eye gaze trajectory during trial *m* and rotation *k*. This is represented by $\varvec{\mathcal {T}} = \{\varvec{\theta }_{i,m,k}(n)\} (m = 1, 2,\ldots , M, k = 1, 2, \dots , K, n = 1, 2,\dots , N_{i,m,k})$. Here, *i* designates each participant and *n* signifies the sequential sampling point, as shown in Fig. 3B.

For a given trial *m* and rotation *k*, the angular velocity $V^{(d)}_{i,m,k}(n)$ in the direction aligned and orthogonal to the optotype movement, which is specified by *d*, was derived by applying a centered difference method-based differential filter^33^. This filter can mitigate the measurement noise when applied to sequentially sampled $\varvec{\theta }_{i,m,k}(n)$. Subsequently, the total gaze movement distance $G^{(d)}_{i,m,k}$ was calculated using the following equation, which served as a metric for assessing eye movement performance:

1 $$\begin{aligned} G^{(d)}_{i,m,k}=\frac{1}{f_s}\sum _{n=1}^{N_{i,m,k}} V^{(d)}_{i,m,k}(n), \end{aligned}$$

where $f_s=500$ is the sampling frequency and $N_{i,m,k}$ represents the total number of samples during a rotation, spanning from when Landolt-C appears to disappear from the screen.

Anisotropy in the DVA and total gaze movement distance were subsequently assessed. Specifically, DVAs derived from the five correctly answered trials were averaged for each participant and the resulting mean values for the horizontal and vertical directions were compared using the paired *t*-test. The direction effect on the total gaze movement distance was analyzed using a linear mixed model expressed by the following Wilkinson-Rogers notation:

2 $$\begin{aligned} G \sim 1+D+S+(D|P) \end{aligned}$$

where *G* denotes the total gaze movement distance. The variable *D* designates the direction in which it adopts a binary representation: $D=0$ corresponds to the horizontal direction and $D=1$ signifies the vertical direction. Thus, the effect size of *D* is interpreted as the difference in the total gaze movement distance between the vertical and horizontal orientations. The term *S* serves as a covariate and represents the optotype speed. *P* is used to differentiate the participants. The term “1” in the model captures the fixed effect associated with the intercept, while the last term denotes the random effects attributed to individual variability in both direction and intercept. The significance level is set at $p<0.001$. Here, if the trajectory $\varvec{\mathcal {T}}$ extends beyond the range of $\varvec{\theta }_{fix,1}$ and $\varvec{\theta }_{fix,5}$ in the major axis direction, it suggests that the head significantly moved from its initial position; therefore, we excluded these trials from the analysis.

### Experimental conditions

The illuminance in the measurement room was 706.3 ± 20.8 lux, and the illumination of the projector was adjusted so that the Landolt-C ring projected on the arc-shaped screen was clearly visible. Specifically, the maximum luminance of the optotype was approximately 2894±50 lux, while its minimum luminance matched that of the measurement room. Consequently, the Michelson contrast of the optotype was in the range of approximately [0.607 and 0.622]. The Landolt-C optotype presented had a diameter measuring 35 mm, accompanied by a gap and line width of 7 mm each. We chose these dimensions to ensure that participants, even those with reduced static visual acuity, could clearly distinguish the optotype, anticipating future clinical applications.

## Results

**Figure 4:**
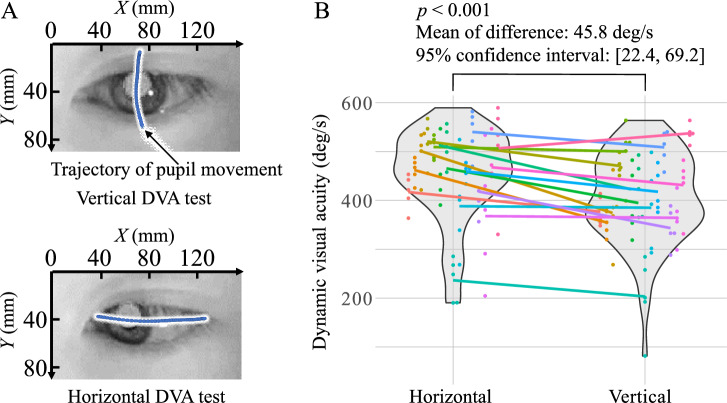
Comparison of vertical and horizontal DVA values. (**A**) displays the examples of measured pupil trajectories with blue dots representing the pupil positions captured in each frame. (**B**) presents a comparison between vertical and horizontal DVA values. The vertical axis denotes DVA, defined as the rotational speed at which a correct answer was given. Different colors are used to distinguish between participants. Each connecting line represents the average horizontal and vertical DVA for individual participants. The results of the paired t-test comparing the average DVA values of each participant are shown above the violin plots.

DVA and eye-movement measurement experiments were conducted according to the protocol described in “Experimental protocol” section. Figure 4A presents examples of measured pupil trajectories, demonstrating both vertical and horizontal eye movements aligned with the respective DVA test directions. Among the participants, nine met the response criterion of five correct answers within five trials, three required six trials, another two required seven trials, and one participant took eight trials to meet the criterion. Figure 4B compares vertical and horizontal DVA. The mean and standard deviation of the DVAs were $451.5 \pm 93.2$ $^\circ$/s and $405.7 \pm 92.4$ $^\circ$/s for the horizontal and vertical directions, respectively. The observed mean difference of 45.8 $^\circ$/s between the horizontal and vertical DVAs was statistically significant ($p < 0.001$), with a $95\%$ confidence interval (CI) ranging from 22.4 to 69.2. This disparity between the two orientations suggests that DVAs are greater in the horizontal direction than in the vertical direction.

**Figure 5:**
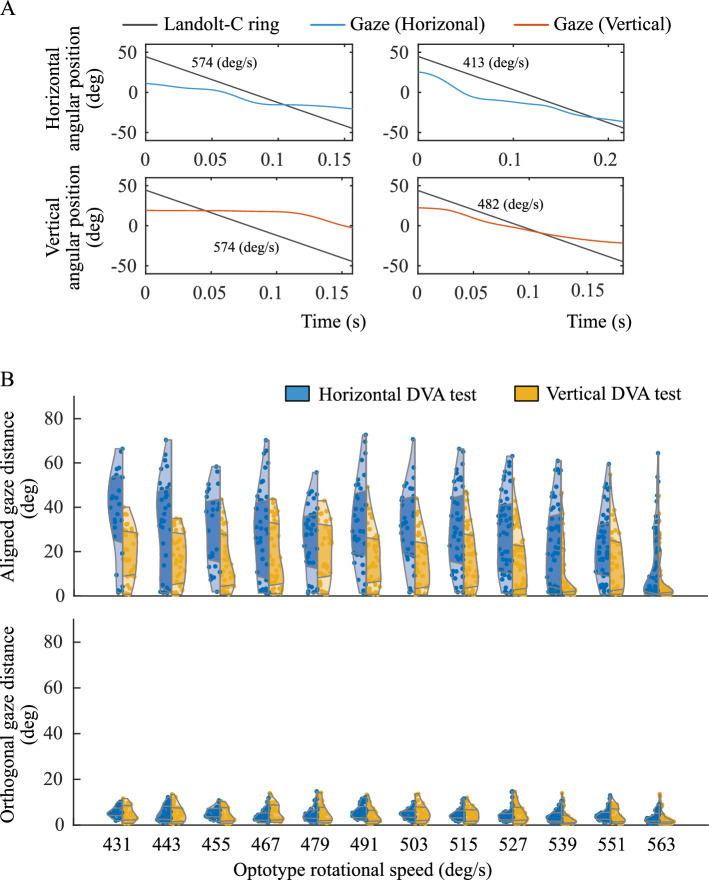
Analysis of gaze movement in horizontal and vertical directions. Panel (**A**) presents the gaze movement overlaid on the trajectory of the Landolt-C ring. The x-axis indicates time, while the y-axis denotes the angular position. The top and bottom rows depict examples from horizontal and vertical DVA tests, respectively. The left and right columns present trajectories from the fastest and slowest Landolt-C movements in a trial, underscoring better gaze tracking with slower Landolt-C movement. Panel (**B**) demonstrates the total gaze movement distance for each Landolt-C rotational speed bin. The upper and lower rows present total gaze movement distance in the direction aligned with and orthogonal to the optotype movement direction. The x-axis represents the minimum value of each speed bin, with each bin spanning 12 deg/s. Vertical movements are shown in blue on the left, and horizontal movements are shown in orange on the right. The shaded regions correspond to the 25th to 75th percentiles. A linear mixed model analysis highlighted a significant difference in total gaze movement distances between vertical and horizontal directions.

We compared $G^{(d)}_{i,m,k}$ between the vertical and horizontal directions, as shown in Fig. 5. Data were obtained from 10 subjects who met the analysis conditions (“Analysis” section). The other five participants might have moved their head positions during the DVA tests, causing the projected pupil position to exceed the upper and lower boundaries of the screen. This suggests that the affine transformation parameter, which was calibrated using the gaze positions at five specific points on the screen, measured at the beginning of the experiment, was invalid for subsequent trials. To prevent this erroneous projection and ensure the validity of our analysis, we excluded these participants from any subsequent analyses that required pupil tracking.

**Table 1:** Directional effect on gaze movement orthogonal to the optotype movement direction: a linear mixed model analysis $$***p < 0.001$$.

	Intercept	Direction	Optotype speed
Fixed effect estimate	17.08	2.961	$-$0.0236
*p* Value	***$1.97 \times 10^{-12}$	0.203	***$1.85 \times 10^{-7}$
Confidence interval	[12.4, 21.8]	[$-$1.69, 7.74]	[$-$0.0325, $-$0.0146]

The lower row of Fig. 5B and Table 1 outline the total gaze movement distance orthogonal to the direction of optotype movement and its corresponding linear mixed model (Eq. (2)) analysis results. A significant intercept, with an effect size of 17.08 $^\circ (p < 0.001, 95\% CI [12.4, 21.8]),$ indicated a minor lateral movement of the pupil. The rotational speed of the optotype had a marginal effect on the lateral movement, although no directional effect was observed. The upper rows of Fig. 5B and Table 2 show the total gaze movement distance aligned with the optotype movement direction, along with the analysis of the results. A significant intercept revealed that the mean total gaze movement distance across the vertical and horizontal DVA tests was 81.07 $^\circ (p < 0.001,$ 95% confidence interval [CI] [71.0, 91.3]). Notably, the rotational speed of the optotype significantly impacts the total gaze movement distance, with an effect size of $-0.108$ ($p < 0.001,$ 95% CI $[-0.122, -0.0939]$). Furthermore, a significant fixed effect of direction was found with an effect size of $-9.35$ ($p <s 0.001,$ 95% CI $[-13.6, -5.09]$). This confirmed that the pupil predominantly moved in alignment with the optotype’s movement. A prominent difference was identified in the total gaze movement distances between the vertical and horizontal directions, with the vertical movement being diminished by 9.35 $^\circ$compared with the horizontal movement.

**Table 2:** Directional effect on gaze movement aligned with the optotype movement direction: a linear mixed model analysis $$***p < 0.001$$.

	Intercept	Direction	Optotype speed
Fixed effect estimate	81.07	$-$11.38	$-$0.1021
*p* Value	***$9.09 \times 10^{-14}$	***$0.159 \times 10^{-3}$	***$2.00 \times 10^{-16}$
Confidence interval	[71.0, 91.3]	[$-$15.7, $-$7.15]	[$-$0.113, $-$0.0909]

## Discussion

The DVA is a pivotal metric for assessing dynamic vision^11^. However, the anisotropy of DVA remains unexplored. To address this gap, we introduced a multidirectional DVA testing device featuring a circular screen. We used this setup to juxtapose the vertical and horizontal DVAs while concurrently recording eye movements. This represents an initial comparative analysis of vertical and horizontal DVAs along with their associated eye movements.

The experimental findings revealed superior DVA performance in the horizontal direction compared with that in the vertical direction. This observation aligns with that of a previous study wherein the vertical DVA was assessed by moving the participant’s head^19–21,25,26^. The comprehensive analysis of pupil trajectories revealed a pronounced proficiency in horizontal gaze movements as compared to their vertical counterparts. These findings point toward the inherent anisotropic properties of gaze movements, which could potentially influence the outcomes of the DVA test.

**Figure 6:**
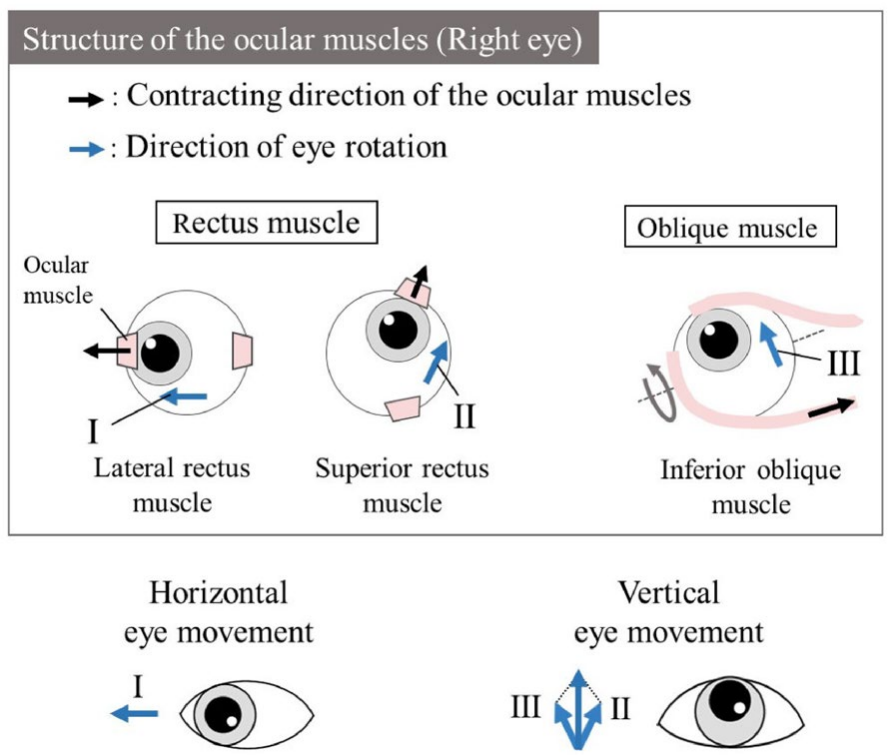
Ocular muscle structure and eye movements.

Several factors may account for the anisotropic characteristics of eye movements. Morphologically, horizontally arranged eyes, because of bilateral anatomical symmetry, may facilitate orientational anisotropy. In addition, the palpebra may interfere with vertical eye movements. When the eyeballs move vertically, the palpebrae need to elevate or depress correspondingly. This was evident when observing the pupil’s position while looking at the top of the screen, which was obscured by the eyelid when gazing at the bottom (Fig. 4B). From an anatomical perspective, as illustrated in Fig. 6, human ocular motions are modulated by two sets of rectus muscles and a singular pair of oblique muscles. The contraction of the rectus muscle causes the eyeball to rotate in the direction corresponding to that contraction. In contrast, the contraction of an oblique muscle instigates rotation in its respective direction but adds a torsional component to the movement. The horizontal ocular shifts are predominantly attributed to contractions of the left and right rectus muscles, symbolized as $(\textrm{I})$ in Fig. 6. Conversely, vertical movements arise from the synergistic action of the superior and inferior rectus muscles, denoted by $(\textrm{II})$, in tandem with the oblique muscles, represented by $(\textrm{III})$. Consequently, vertical eye movements may require more nuanced muscular coordination compared to their horizontal counterparts^34^. Further, neurophysiologically, the retina of each eye is divided into two halves (left and right) in a lateralized arrangement. This division allows each eye to capture more space on one side (e.g., the left visual field for the right eye and vice versa) than on the other. This asymmetric visual capture may contribute to preferential sensitivity to horizontal movements, possibly explaining the observed anisotropy. In addition, from an evolutionary perspective, Homo sapiens, an erect bipedal species, tends to focus more on the horizontal plane. This inclination likely has its roots in our ancestors’ need for vigilance against terrestrial predators (e.g., lions) rather than aerial threats (e.g., birds). Such biological conditioning towards the horizontal plane could further explain the supremacy of horizontal DVA, reflecting an adaptive advantage that has persisted throughout human evolution. A more comprehensive examination of these factors is essential for understanding the underlying causes of the directional characteristics of eye movements.

One limitation of our proposed device is the difficulty in controlling luminance and contrast during DVA measurements. The method used to present a flickerless image restricts the optotype to a binary representation, rendering it challenging to precisely adjust luminance levels. Although a glass-neutral density filter has been suggested as a possible solution, its effectiveness requires verification. Future research should address this limitation to improve the device’s ability to perform more comprehensive DVA assessments. In addition, examining the interaction between the gap direction of the Landolt-C and its rotational direction, as well as its relationship with the operation direction of the response device, could help elucidate the interplay between visual recognition and bodily reactions.

## Conclusion

In this study, we developed a system tailored to gauge multidirectional DVA. Our experiments, which encompassed both vertical and horizontal DVA evaluations, showed superior performance in the horizontal DVA compared to its vertical counterpart. A subsequent eye movement assessment revealed an extended total gaze movement distance in the horizontal trajectory compared with its vertical counterpart. This observed trend may be intricately linked to the anisotropic nature of DVA performance.

The genesis of our interest in this topic stems from sports like volleyball and baseball, where athletes are often tasked with vertically tracking fast-moving balls. The empirical evidence from our study underscores the inherent challenge of tracking vertical rather than horizontal movements. As a logical extension, we hypothesized that tailored training regimes aimed at bolstering vertical motion tracking might enhance athletic performance. Our future research endeavors will evaluate this potential, seeking to bridge the understanding of DVA’s role and its potential applications across diverse sports disciplines.

## Data Availability

The datasets generated and/or analyzed in the current study are not publicly available because of the lack of explicit data-sharing permission from the participants. The study was conducted under strict ethical guidelines, and the participants’ informed consent did not include provisions for the public sharing of their data. However, the datasets can be made available from the corresponding author upon reasonable request, subject to the ethical considerations and limitations imposed by consent agreements.
