# Author Correction: Vertical dynamic visual acuity is significantly lower than horizontal dynamic visual acuity

**DOI:** 10.1038/s41598-024-52055-x

**Published:** 2024-01-22

**Authors:** Aoi Tachihara, Zu Soh, Tomohiko Mizuguchi, Akihiko Kandori, Seiji Hama, Toshio Tsuji

**Affiliations:** 1 Electrical, Systems, and Control Engineering Program, Graduate School of Advanced Science and Engineering, Hiroshima University, Higashi-Hiroshima, Hiroshima 739-8527 Japan; 2 New Business Producing Division, Maxell Ltd., Yokohama, 240-0005 Japan; 3 Center for Exploratory Research, Research and Development Group, Hitachi. Ltd., Tokyo, 185-8601 Japan; 4 Department of Rehabilitation, Hibino Hospital, Hiroshima, 731-3164 Japan; 5 Department of Neurosurgery, Center for Brain, Mind and KANSEI Sciences Research, Hiroshima University, Hiroshima, 734-8553 Japan

Correction to: *Scientific Reports* 10.1038/s41598-023-48292-1, published online 28 November 2023

The Funding section in the original version of this Article was omitted. The Funding section now reads:

"Hitachi Ltd has funded the Article Processing Charges for this publication."

The original Article has been corrected.

